# Distribution of *ace-1*^*R *^and resistance to carbamates and organophosphates in *Anopheles gambiae s.s. *populations from Côte d'Ivoire

**DOI:** 10.1186/1475-2875-9-167

**Published:** 2010-06-16

**Authors:** Ludovic P Ahoua Alou, Alphonsine A Koffi, Maurice A Adja, Emmanuel Tia, Philippe K Kouassi, Moussa Koné, Fabrice Chandre

**Affiliations:** 1 Institut Pierre Richet (IPR), BP 47 Abidjan, Côte d'Ivoire; 2 Laboratoire de Zoologie et Biologie Animale, Université de Cocody, 22 BP 582 Abidjan 22, Côte d'Ivoire; 3 Centre d'Entomologie Médicale et Vétérinaire (CEMV), Université de Bouake, 27 BP 529 Abidjan 27, Côte d'Ivoire; 4 Institut de Recherche pour le Développement (IRD)/Laboratoire de Lutte Contre les Insectes Nuisibles (LIN) LIN, IRD/UR 016, 911 Ave Agropolis, 34394 Montpellier Cedex 5, France

## Abstract

**Background:**

The spread of pyrethroid resistance in *Anopheles gambiae s.s. *is a critical issue for malaria vector control based on the use of insecticide-treated nets. Carbamates and organophosphates insecticides are regarded as alternatives or supplements to pyrethroids used in nets treatment. It is, therefore, essential to investigate on the susceptibility of pyrethroid resistant populations of *An. gambiae s.s. *to these alternative products.

**Methods:**

In September 2004, a cross sectional survey was conducted in six localities in Côte d'Ivoire: Toumbokro, Yamoussoukro, Toumodi in the Southern Guinea savannah, Tiassalé in semi-deciduous forest, then Nieky and Abidjan in evergreen forest area. *An. gambiae *populations from these localities were previously reported to be highly resistant to pyrethroids insecticides. Anopheline larvae were collected from the field and reared to adults. Resistance/susceptibility to carbamates (0.4% carbosulfan, 0.1% propoxur) and organophosphates (0.4% chlorpyrifos-methyl, 1% fenitrothion) was assessed using WHO bioassay test kits for adult mosquitoes. Then, PCR assays were run to determine the molecular forms (M) and (S), as well as phenotypes for insensitive acetylcholinesterase (AChE1) due to G119S mutation.

**Results:**

Bioassays showed carbamates (carbosulfan and propoxur) resistance in all tested populations of *An. gambiae s.s. *In addition, two out of the six tested populations (Toumodi and Tiassalé) were also resistant to organophosphates (mortality rates ranged from 29.5% to 93.3%). The M-form was predominant in tested samples (91.8%). M and S molecular forms were sympatric at two localities but no M/S hybrids were detected. The highest proportion of S-form (7.9% of *An. gambiae *identified) was in sample from Toumbokro, in the southern Guinea savannah. The G119S mutation was found in both M and S molecular forms with frequency from 30.9 to 35.2%.

**Conclusion:**

This study revealed a wide distribution of insensitive acetylcholinesterase due to the G119S mutation in both M and S molecular forms of the populations of *An. gambiae s.s. *tested. The low cross-resistance between carbamates and organophosphates highly suggests involvement of other resistance mechanisms such as metabolic detoxification or F290V mutation.

## Background

Malaria vectors control mainly relies on the use of insecticide-treated nets (ITN) and indoor residual spraying (IRS). Pyrethroids are the only group of insecticides currently recommended for net treatment [1]. Although pyrethroid resistance in the most important malaria vector *Anopheles gambiae s.s. *has become widespread in several African countries [2-5], field studies in experimental huts and at community level using malaria indicators have shown that pyrethroid-treated bed nets remain usually effective against pyrethroid resistant mosquitoes [6-9]. However, the evolution of pyrethroid resistance in *An. gambiae s.s. *represent a threat for malaria control. To prevent any significant decline of the efficiency of pyrethroids, harmful to the malaria control, management strategies of pyrethroid resistance are envisaged through the exploration of news tools or combination of existing ones.

One of these strategies, used in agriculture as well as in public health, consists to associate in the same treatment, several molecules having different modes of action. Although developed initially for agricultural use and for indoor residual spraying, carbamates and organophosphates constitute a new prospect to circumvent pyrethroid resistance in *An. gambiae s.s.*

In area of high prevalence of *kdr *in Côte d'Ivoire, experimental hut trials of carbamates or organophosphates alone and in combination with pyrethroids on mosquito nets showed very promising results [8,10-13]. However, little is known about the susceptibility status of pyrethroid resistance populations of *An. gambiae *to organophosphates and carbamates in Côte d'Ivoire, as well as potential resistance mechanisms.

Acetylcholinesterase (AChE) is a common target for carbamates and organophosphates. These insecticides blocks transmission of nerve impulses by irreversible inhibition of AChE at cholinergic synapses, causing insect death. Cross-resistance to carbamates and organophosphates can arise by insensitive AChE mechanism due to the glycine to serine substitution (G119S mutation) resulting from a single point mutation in the *ace-1 *gene [14]. The G119S mutation was selected independently in several mosquitoes species including *An. gambiae s.s.*, the major malaria vector in Africa [11,14-17]. This mutation was found in both M and S molecular forms of *An. gambiae *from Côte d'Ivoire [16,17].

In the current study, the geographic extent of insensitive AChE mechanism in *An. gambiae s.s. *populations from Côte d'Ivoire according to molecular forms, as well as their susceptibility status to carbamates and organophosphates were investigated.

## Methods

### Mosquito populations and sampling sites

**Figure 1:**
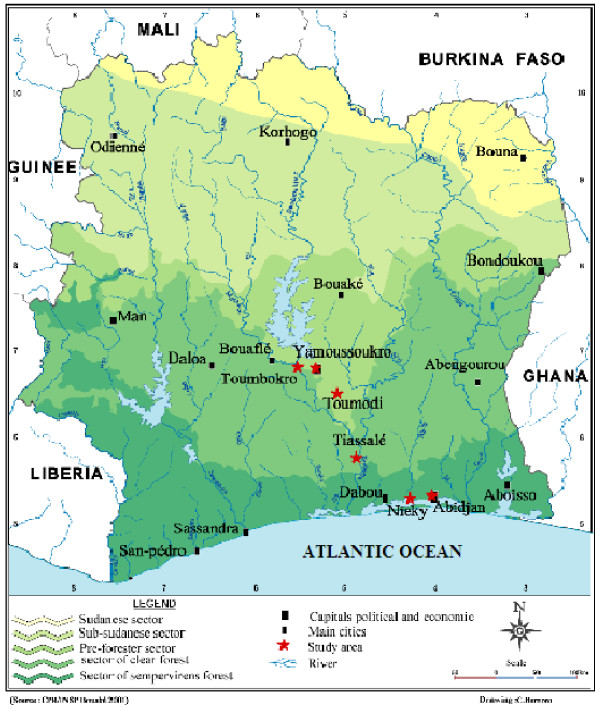
Map of Côte d'Ivoire showing the localities in the different ecological zones where anopheline mosquitoes were collected

The study sites form a north-to-south transect across the Southern Guinea savannah, the semi-deciduous forest and the evergreen forest areas in Côte d'Ivoire. The last two zones are characterized by intensive human activities and agricultural land-degraded forest mosaic. Mosquitoes were collected during the rainy season from six localities: Toumbokro (7°N; 5°35' W), Yamoussoukro (6°82' N; 5°28' W) and Toumodi (6°55' N; 5°03' W) located in the Southern Guinea savannah, Tiassalé (5°88' N; 4°38' W) in a semi-deciduous forest area, then Nieky (5°20'N; 4°10'W) and Abidjan (5°33'N; 4°03' W) in a evergreen forest area (Figure 1). Samples were collected from coffee and cocoa industrial plantations in Toumbokro, banana cultivation fields in Nieky and in urban areas in Yamoussoukro, Toumodi, Tiassalé and Abidjan. Mosquitoes were collected at larval stage, brought to the laboratory and reared until for emergence of adults. A reference laboratory strain of *An. gambiae s.s. *named "Kisumu", native from Kenya and susceptible to all insecticides was used as control.

### Susceptibility test

Bioassays were carried out using WHO test kits for adults mosquitoes [18] with four insecticides of technical grade quality: two carbamates (0.4% carbosulfan, 0.1% propoxur) and two organophosphates (0.4% chlorpyrifos-methyl and 1% fenitrothion). Filter papers were impregnated according to WHO specifications by the Institut Pierre Richet de Bouaké. Papers were stored at 4°C and were not used more than three times.

Tests were performed with batches of 25 unfed females of *An. gambiae s.s.*, 2-5 days old, four replicates per insecticide. Mosquitoes were exposed to the insecticide treated papers for 60 min at 27 ± 1°C and 80% relative humidity. After exposure, mosquitoes were kept in observation tubes, supplied with 10% honey solution and held for 24 h before scoring mortality. Batches exposed to untreated papers were used as control.

### M/S taxon determination

According to previous studies, *An. gambiae *complex in Côte d'Ivoire was only represented by *An. melas *on the Atlantic littoral area and *An. gambiae s.s.*, the most widespread all over the country [19-21]. So the PCR analysis in this study was carried directly on the molecular forms of *An. gambiae s.s.*. Genomic DNA was extracted from individual mosquitoes according to Collins *et al *[22] and used for PCR analysis to determine M/S taxon according to Favia *et al *[23]. The PCR conditions were 10 min at 94°C as initial step, followed by 29 cycles (94°C for 30 seconds, 63°C for 30 seconds and 72°C for 30 seconds). After the last cycle the products were finally extended for 7 min at 72°C. Primers used in the PCR were: R5 5'GCCAATCCGAGCTGATAGCGC3', R3 5'CGAATTCTAGGGAGCTCCAG3', Mopint 5'GCCCCTTCCTCGATGGCAT3', B/S 5'ACCAAGATGGTTCGTTGC3'. Amplified fragments were analysed on a 1.5% agarose gel.

### DNA diagnostic test for insensitive acetylcholinesterase G119S mutation

Genomic DNA extracted from the field samples and used for PCR was also used to determine the phenotypes for insensitive AChE G119S mutation according to Weill *et al *[16]. The DNA was PCR amplified with the primers Ex3Agdir 5'GATCGTGGACACCGTGTTCG3' and Ex3Agrev 5'AGGATGGCCCGCTGGAACAG3' for an initial denaturation step of 3 min at 94°C, followed by thirty-five cycles (94°C for 30 seconds, 62°C for 30 seconds and 72°C for 20 seconds). After the final cycle the products were extended for 5 min at 72°C. The PCR fragments were then digested with *Alu *I restriction enzyme and fractionated on a 2% agarose gel. The two primers produced a 403 bp fragment, which is undigested by *Alu*I for susceptible homozygous mosquitoes (SS), and cut into two fragments (253 bp and 150 bp) for homozygous resistant (RR). Heterozygous individuals (RS) display a combined pattern.

### Data analysis

Mortality data were analysed according to WHO [18]. To compare the status of insecticide resistance, Fisher's exact test was performed to determine if there was any significant difference between mortality rates of two given populations of *An. gambiae s.s. *using Statistica 6.0. Allelic frequencies of G119S mutation were analysed using the version 3.2a of Genepop [24]. To assess if the mutation frequencies was identical across populations, the test of genotypic differentiation was performed [25].

## Results

### Susceptibility to carbamates and organophosphates

Mortality rates of the Kisumu reference strain to all insecticides was 100% (Table 1). Conversely, all the field samples were resistant to carbamates, with mortalities rates less than 83%. Susceptibility to chlorpyrifos-methyl was assessed on five populations except on the Yamoussoukro population. Chlorpyrifos-methyl resistance was detected in Toumodi and Tiassalé, with 82-94% mortality rates, while it was suspected in Toumbokro with 97% mortality rate. The two other populations were fully susceptible to this organophosphate. Fenitrothion resistance was observed in five out of the six populations tested (Toumbokro, Toumodi, Tiassalé, Nieky, Abidjan). Only the Yamoussoukro population was fully susceptible to this insecticide. Overall the two populations from Toumodi and Tiassalé were resistant to all insecticides used. Tiassalé sample was the most resistant to carbamates and organophosphates with mortality rates of 3% and 12% for carbosulfan and propoxur and 83% and 30% for chlorpyrifos-methyl and fenitrothion, respectively.

**Table 1:** Mortality of a susceptible strain (Kisumu) and wild populations of *Anopheles gambiae s.s*. exposed to diagnostic doses of technical material of insecticides

	Carbosulfan (0.4%)		Propoxur (0.1%)		Chlorpyrifos-methyl (0.4%)		Fenitrothion (1%)	
	
Localities	Mort	Status	Mort	Status	Mort	Status	Mort	Status
Kisumu	100 (101)	S	100 (104)	S	100 (104)	S	100 (102)	S
Toumbokro	21.4^b ^(98)	R	40.9^b ^(88)	R	96.9 (97)	S	89.5^bc ^(105)	R
Yamoussoukro	42.3^d ^(104)	R	69.7^c ^(99)	R	NT		99.0 (98)	S
Toumodi	28.4^bc ^(95)	R	56.3^c ^(96)	R	93.3^b ^(89)	R	81.1^b ^(95)	R
Tiassalé	2.8^a ^(217)	R	11.6^a ^(224)	R	82.8^a ^(192)	R	29.5^a ^(207)	R
Nieky	28.2^bc ^(103)	R	82.8^d ^(99)	R	100 (102)	S	95.7^c ^(92)	R
Abidjan	39.0^cd ^(100)	R	58.4^c ^(101)	R	100 (102)	S	96.0^c ^(99)	R

Number of tested mosquitoes in parentheses; NT: no tested; Mort: Mortality rate 24 h post exposure; S: indicates susceptibility; R: suggests resistance.

NB: Numbers in the same column with the same superscript do not differ significantly by Fisher's exact test (p > 0.05)

### Molecular forms and frequencies of the G119S mutation

All PCR analysis to determine M/S molecular forms realized in this study were positive, showing either the form M or the form S. So it was not necessary to make the PCR analysis for species identification [26].

Three hundred twenty-eight mosquitoes were identified to molecular forms and analyzed for the G119S mutation; results are shown in Table 2. The M and S molecular forms of *An. gambiae s.s. *occurred in sympatry in two of the six localities, namely Toumbokro and Toumodi in the savannah area. However, the M-form was predominant in the six areas, representing 91.8% of the whole sample (n = 328). In sympatric areas, the frequencies of the S-form were 41.9% (n = 62) and 1.3% (n = 76) respectively in Toumbokro and Toumodi. However, no M/S heterozygote was found.

**Table 2:** Acetylcholinesterase phenotypes and frequency of G119S mutation in the molecular M and S forms of *Anopheles gambiae s.s*

Locality	M Form					S Form				
		
	Phenotypes					Phenotypes				
						
	S	RS	R	F(G119S)%	(%)*	S	RS	R	F(G119S)%	(%)*
Toumbokro	12	24	0	**33.3 **^**b**^	58.1	8	18	0	**34.6**	41.9
Yamoussoukro	7	24	0	**38.7 **^**b**^	100	0	0	0	**_**	0
Toumodi	47	28	0	**18.6 **^**a**^	98.7	0	1	0	**50.0**	1.3
Tiassalé	0	82	0	**50.0 **^**c**^	100	0	0	0	**_**	0
Nieky	23	7	0	**11.7**^**a**^	100	0	0	0	**_**	0
Abidjan	26	21	0	**22.3**^**a**^	100	0	0	0	**_**	0
Total	115	186	0	**30.9**	91.8	8	19	0	**35.2**	8.2

*: Percentage of each molecular form in the population tested

^a,b ^Values sharing a superscript letter are not significantly different at the 5% level for G119S mutation distribution

The G119S mutation was detected in all the six populations tested, but only at heterozygote state, either in the M or in the S form. The highest mutation frequency was observed in the M form from the Tiassalé urban area located in semi-deciduous forest (50%) and the lowest in the M form from the Nieky banana cultivation fields in evergreen forest (12%). No significant difference was seen between G119S mutation frequencies in M and S forms from Toumbokro (p = 0.9153).

## Discussion

The distribution of M and S molecular forms of *An. gambiae *s.s. in the study agrees with previous findings that reported both M and S forms in Guinea savannah areas and only the M form in the forest areas [27-30]. This geographic distribution seems to follow more the global environment than the breeding sites nature. Both forms are involved in carbamate and organophosphate resistance, although at different level according to insecticides. Indeed, in this study, *An. gambiae s.s. *displayed large variations in resistance level to carbamates and organophosphates. Although the wild populations were all resistant to carbamates, resistance was less marked to propoxur than to carbosulfan at WHO diagnostic concentrations.

All these populations were as resistant to carbosulfan as the population of Yaokoffikro in surrounding area of Bouaké [11]. The resistance reported in Bouaké was attributed to agricultural or domestic hygiene or public health use of carbamates. In Burkina-Faso, Diabaté *et al *[31] attributed *An. gambiae s.s. *pyrethroid resistance in cotton field areas to their use in agriculture.

The observed cross-resistance to organophosphates and carbamates in Tiassalé and Toumodi highlights implication of their common target site: the AChE-1. Although the mutation *ace-1 *G119S provided cross-resistance to organophosphates and carbamates, the resistance level greatly varied between both insecticide families. This difference observed in resistance level could be the consequence of their difference observed in dominance level relied on insecticide specificity. According to Djogbénou *et al *[32], dominance status of *ace-1 *G119S varied between semirecessivity with fenitrothion and chlorpyrifos methyl to semidominance with propoxur and carbamates. The fact that low cross-resistance was observed in the other populations, suggests and confirms potential involvement of metabolic resistance mechanisms and/or alternative mutation associated to G119S. This may explain why mortality rates to organophosphates among samples from Nieky, Abidjan and Yamoussoukro were so strong despite confirmed resistance level to carbamates in bioassays.

Such result could also be explained by possible cross-resistance between organophosphates and pyrethroids based on an increased detoxification mechanism were as suggested for other anopheline species selected for pyrethroid resistance [33].

Moreover an alternative mutation in *ace-1 *gene was described in the *Culex pipiens *strain originating from Cyprus. This mutation is F290V substitution and it confers cross-resistance to OP and carbamate insecticides [34]. Because *C. pipiens *and *An. gambiae s.s. *share G119S, it is possible that they share also this other mutation. Asidi *et al *[13] had noted that G119S mutation did not confer effective resistance to chlorpyrifos-methyl. Yet, the G119S mutation involved certainly a high resistance to carbamate but could enhance organophosphate hydrolysis. Similar mutations in a homologous position to G119S are known to alter substrate specificity in *Drosophila melanogaster *and enhanced hydrolysis of some organophosphates [35].

The presence of G119S mutation in both M and S forms of *An. gambiae s.s. *has already been reported by Weill *et al *[16] and Djogbénou *et al *[17] and was suggested to result from introgression between forms. The wide distribution of *ace-1*^*R *^reported here could result from an unique event that then spread as reported in *C. pipiens *amplified esterase B2 genes through the world [36].

The absence of homozygous resistant individuals might be related to high fitness cost of the G119S mutation, resulting on death of the homozygous resistant [13,16,17]. Indeed, greater mortality of resistant individuals during pupation relative to their sensitive counterparts was reported. There was also evidence for costs to adult fitness as resistant individuals were smaller than sensitive adults [37]. Consequently, in area where the resistant allele *ace-1*^*R *^is present, resistant mosquitoes will mainly at heterozygote state (*ace-1*^*RS*^). Because of this fitness cost, at least one duplication combining resistant and susceptible alleles of the *ace-1 *locus has recently appeared, started to spread and replace *ace-1*^*R *^in treated areas [17,38-41]. Duplications lead to an excess of heterozygotes in natural populations because that heterozygotes involving either *ace-1*^*S *^or *ace-1*^*R *^alleles do not exhibit deleterious side effects. To date, no specific test is available for detecting specifically *ace-1 *duplications as mosquitoes carrying duplications appear as heterozygous for *ace-1*^*R *^mutation.

Further investigation is needed to tackle the origin of the difference of resistance between carbamates and organophosphates.

## Conclusion

Data from this study complemented resistance to carbamates and organophosphates in *An. gambiae s.s. *populations from Côte d'Ivoire and the wide distribution of G119S mutation in both molecular forms. The low cross-resistance between carbamates and organophosphates through susceptibility tests in most of the populations suggests the involvement of other resistance mechanisms, probably a metabolic detoxification or an alternative mutation such as the F290V substitution. These results must be carefully considered while elaborating malaria control programs in Côte d'Ivoire.

## Competing interests

The authors declare that they have no competing interests.

## Authors' contributions

LPAA, AAK designed the study, conducted the field work, genotyping, summarized the data and drafted the manuscript. MAA, ET jointly carried out PCR assays, and interpreted the results. PKK and MK supervised LPAA and AAK and contributed to the manuscript. FC contributed to design of the study and manuscript drafting. All authors read and approved the final manuscript.

## References

[B1] Guillet P Chandre F Mouchet J L'utilisation des insecticides en santé publique: état et perspectives Med Mal Infect 1997 27 552 557 10.1016/S0399-077X(97)80117-5

[B2] Elissa N Mouchet J Riviere F Meunier JY Yao K Resistance of *Anopheles gambiae s.s. *to pyrethroids in Côte-d'Ivoire Ann Soc Belge Med Trop 1993 73 291 294 8129474

[B3] Chandre F Darriet F Manga L Akogbeto M Faye O Mouchet J Guillet P Status of pyrethroid resistance in *Anopheles gambiae s.l.* Bull World Health Organ 1999 77 230 234 10212513 PMC2557627

[B4] Chandre F Darriet F Manguin S Brengues C Carnevale P Guillet P Pyrethroid cross resistance spectrum among population of *Anopheles gambiae s.s. *from Côte d'Ivoire J Am Mosq Control Assoc 1999 15 53 59 10342269

[B5] Ranson H Jensen B Vulule JM Wang X Hemingway J Collins FH Identification of a point mutation in the voltage-gated sodium channel gene of Kenyan *Anopheles gambiae s.s. *associated with resistance to DDT and pyrethroids Ins Mol Biol 2000 9 491 497 10.1046/j.1365-2583.2000.00209.x 11029667

[B6] Darriet F N'Guessan R Koffi AA Konan LY Doannio JMC Chandre F Carnevale P Impact de la résistance de *Anopheles gambiae s.s. *aux pyréthrinoïdes sur l'efficacité des moustiquaires imprégnées dans la prévention du paludisme: résultats des essais en cases expérimentales avec la deltaméthrine Bull Soc Pathol Exot 2000 93 131 134 10863621

[B7] N'Guessan R Darriet F Doannio JMC Chandre F Carnevale P Olyset Net^® ^efficacy against pyrethroid-resistant *Anopheles gambiae s.s. *and *Culex quinquefasciatus *after 3 years' field use in Cote d'Ivoire Med Vet Entomol 2001 15 97 104 10.1046/j.1365-2915.2001.00284.x 11297108

[B8] Hougard JM Corbel V N'Guessan R Darriet F Chandre F Akogbeto M Baldet T Guillet P Carnevale P Traoré-Lamizana M Efficacy of mosquito nets treated with insecticide mixtures or mosaics against insecticide resistant *Anopheles gambiae s.s. *and *Culex quinquefasciatus *(Diptera: Culicidae) in Cote d'Ivoire Bull Entomol Res 2003 93 491 498 10.1079/BER2003261 14704095

[B9] Henry Mc Assi Sb Rogier C Dossou-Yovo J Chandre F Guillet P Carnevale P Protective efficacy of lambda-cyhalothrin treated nets in *Anopheles gambiae *pyrethroid resistance areas of Cote d'Ivoire Am J Trop Med Hyg 2005 73 859 864 16282294

[B10] Guillet P N'Guessan R Darriet F Traore-Lamizana M Chandre F Carnevale P First trials of combined pyrethroid and carbamate treated mosquito nets active against pyrethroid resistant *Anopheles gambiae s.s. *and *Culex quinquefasciatus* Med Vet Entomol 2001 15 105 112 10.1046/j.1365-2915.2001.00288.x 11297094

[B11] N'guessan R Darriet F Guillet P Carnevale P Traore-Lamizana M Corbel V Koffi AA Chandre F Resistance to carbosulfan in *Anopheles gambiae s.s. *from Ivory Coast based on reduced sensitivity of acetylcholinesterase Med Vet Entomol 2003 17 1 7 10.1046/j.1365-2915.2003.00406.x 12680920

[B12] Asidi AN N'Guessan R Hutchinson RA Traore-Lamizana M Carnevale P Curtis CF Experimental hut comparisons of nets treated with carbamate or pyrethroid insecticides, washed or unwashed, against pyrethroid-resistant mosquitoes Med Vet Entomol 2004 18 134 140 10.1111/j.0269-283X.2004.00485.x 15189238

[B13] Asidi AN N'Guessan R Koffi AA Curtis CF Hougard JM Chandre F Darriet F Zaim M Rowland MW Experimental hut evaluation of bednets treated with an organophosphate (chlorpyrifos-methyl) or a pyrethroid (lambdacyalothrin) alone and in combination against insecticide-resistant *Anopheles gambiae s.s. *and *Culex quinquefasciatus *mosquitoes Malar J 2005 4 25 10.1186/1475-2875-4-25 15918909 PMC1156935

[B14] Weill M Lutfalla G Mogensen K Chandre F Berthomieu A Berticat C Pasteur N Philips A Fort P Raymond M Insecticide resistance in mosquito vectors Nature 2003 423 136 137 10.1038/423136b 12736674

[B15] Bourguet D Capela R Raymond M An insensitive acetylcholinesterase in *Culex pipiens *L. mosquitoes from Portugal J Econ Entomol 1996 89 1060 1066 8913110 10.1093/jee/89.5.1060

[B16] Weill M Malcolm C Chandre F Mogensen K Berthomieu A Marquine M Raymond M The unique mutation in *Ace-1 *giving high insecticide resistance is easily detectable in mosquito vectors Insect Mol Biol 2004 13 1 7 10.1111/j.1365-2583.2004.00452.x 14728661

[B17] Djogbénou L Chandre F Berthomieu A Dabiré R Koffi A Alout H Weill M Evidence of introgression of the *ace-1R *mutation and of the *ace-1 *duplication in West African *Anopheles gambiae *s.s Plos ONE 2008 3 5 e2172 10.1371/journal.pone.0002172 18478097 PMC2377098

[B18] WHO Tests procedures for insecticide resistance monitoring in malaria vectors, bio-efficacy and persistence of insecticides on treated surfaces 1998 12 WHO/CDS/CPC/MAL/

[B19] Doucet J Adam JP Binson G Les Culicidae de la Côte d'Ivoire Ann Parasitol Hum Comparée 1960 25 390 408 13724040

[B20] Koffi AA Chandre F Tia E Darriet F Touré M Dossou-Yovo J N'guessan R Konan YL Doannio JMC Carnevale P Pyrethroid resistance in populations of *An. gambiae *s.l. from Côte d'Ivoire Insecticide Resistance in Malaria Vectors, Multilateral Initiative on Malaria 2001 Harare, Zimbabwe

[B21] Koffi AA Résistance d'*Anopheles gambiae *(Gilles, 1902) aux pyréthrinoïdes et son impact sur la lutte antivectorielle, par les moustiquaires imprégnées d'insecticides Thèse de Doctorat 2002 Université de Cocody, UFR Biosciences, Abidjan, Côte d'Ivoire

[B22] Collins FH Finnerty V Petrarca V Ribosomal DNA probes differentiate five cryptic species in the *Anopheles gambiae s.s. *complex Parasitology 1988 30 231 240 3271987

[B23] Favia G Lanfrancotti A Spanos L Siden-Kiamos I Louis C Molecular characterization of ribosomal DNA polymorphisms discriminating among chromosomal forms of *Anopheles gambiae s.s.* Insect Mol Biol 2001 10 19 23 10.1046/j.1365-2583.2001.00236.x 11240633

[B24] Raymond M Rousset F Genepop (version 1.2), population genetics software for exact tests and eucumenicism J Heredity 1995 86 248 249

[B25] Goudet J Raymond M De Meeüs T Rousset F Testing differentiation in diploid populations Genetics 1996 144 1933 1940 8978076 10.1093/genetics/144.4.1933 PMC1207740

[B26] Scott J Brogdon W Collins F Identification of single specimens of the *Anopheles gambiae *complex by PCR Am J Trop Med Hyg 1993 49 520 529 8214283 10.4269/ajtmh.1993.49.520

[B27] della Torre A Fanello C Akogbeto M Dossou-Yovo J Favia G Petrarca V Coluzzi M Molecular evidence of incipient speciation within *Anopheles gambiae s.s. *in West Africa Insect Mol Biol 2001 10 9 18 10.1046/j.1365-2583.2001.00235.x 11240632

[B28] della Torre A Tu Z Petrarca V On the distribution and genetic differentiation of *Anopheles gambiae s.s. *molecular forms Insect Biochem Mol Biol 2005 35 755 769 10.1016/j.ibmb.2005.02.006 15894192

[B29] Fanello C Petrarca V della Torre A Santolamazza F Dolo G Coulibaly M Alloueche A Curtis CF Toure YT Coluzzi M The pyrethroid knock-down resistance gene in the *Anopheles gambiae *complex in Mali and further indication of incipient speciation within *An. gambiae s.s.* Insect Mol Biol 2003 12 241 245 10.1046/j.1365-2583.2003.00407.x 12752657

[B30] Onyabe DY Vajime CG Nock IH Ndams IS Akpa AU Alaribe AA Conn JE The distribution of M and S molecular forms of Anopheles gambiae in Nigeria Trans R Soc Trop Med Hyg 2003 97 605 608 10.1016/S0035-9203(03)80045-7 15307438

[B31] Diabaté A Baldet T Chandre F Akogbeto M Guiguemde TR Darriet F Brengues C Guillet P Hemingway J Small GJ Hougard JM The role of agricultural use of insecticides in resistance to pyrethroids in *Anopheles gambiae s.l. *in Burkina Faso Am J Trop Med Hyg 2002 67 617 622 12518852 10.4269/ajtmh.2002.67.617

[B32] Djogbénou L Weill M Hougard JM Raymond M Akogbéto M Chandre F Characterization of insensitive acetylcholinesterase (*ace-1*^*R*^) in *Anopheles gambiae *(Diptera: Culicidae): Resistance levels and dominance J Med Entomol 2007 44 805 810 10.1603/0022-2585(2007)44[805:COIAAI]2.0.CO;2 17915512

[B33] Brogdon WG Barber AM Fenitrothion-deltamethrin cross-resistance conferred by esterases in Guatemalan *Anopheles albimanus* Pest Biochem Phys 1987 37 130 139 10.1016/0048-3575(90)90118-L

[B34] Alout H Berthomieu A Hadjivassilis A Weill M A new amino-acid substitution in acetylcholinesterase 1 confers insecticide resistance to *Culex pipiens *mosquitoes from Cyprus Insect Biochem Mol Biol 2007 37 41 47 10.1016/j.ibmb.2006.10.001 17175445

[B35] Newcomb RD Campbell PM Ollis DL Cheah E Russel RJ Oakeshott JG A single amino acid substitution converts a carboxylesterase to an organophosphorus hydrolase and confers insecticide resistance on blowfly Proc Natl Acad Sci USA 1997 94 7464 7468 10.1073/pnas.94.14.7464 9207114 PMC23844

[B36] Raymond M Callaghan A Fort P Pasteur N Worldwide migration of amplified insecticide resistance genes in mosquitoes Nature 1991 350 151 153 10.1038/350151a0 2005964

[B37] Djogbénou L Noel V Agnew P Costs of insensitive acetylcholinesterase insecticide resistance for the malaria vector *Anopheles gambiae *homozygous for the G119S mutation Malar J 2010 9 12 10.1186/1475-2875-9-12 20070891 PMC2816975

[B38] Lenormand T Guillemaud T Bourguet D Raymond M Appearance and sweep of a gene duplication: Adaptive response and potential for new functions in the mosquito *Culex pipiens* Evolution 1998 52 1705 1712 10.2307/2411343 28565319

[B39] Labbé P Berthomieu A Berticat C Alout H Raymond M Lenormand Weill M Independent duplications of the acetylcholinesterase gene conferring insecticide resistance in the mosquito *Culex pipiens* Mol Biol Evol 2007 24 1056 1067 10.1093/molbev/msm025 17283366

[B40] Labbé P Berticat C Berthomieu A Unal S Bernard C Weill M Lenormand T Forty years of erratic insecticide resistance evolution in the mosquito *Culex pipiens* PLOS Genet 2007 3 2190 2199 10.1371/journal.pgen.0030205 PMC2077897 18020711

[B41] Djogbénou L Labbé P Chandre F Pasteur N Weill M Ace-1 duplication in *Anopheles gambiae: *a challenge for malaria control Malar J 2009 8 70 10.1186/1475-2875-8-70 19374767 PMC2679766

